# Data for induction of cytotoxic response by natural and novel quercetin glycosides

**DOI:** 10.1016/j.dib.2015.11.066

**Published:** 2015-12-12

**Authors:** Alexis H. Haskins, Cathy Su, Anya Engen, Victoria A. Salinas, Junko Maeda, Mitsuru Uesaka, Yasushi Aizawa, Takamitsu A Kato

**Affiliations:** a Department of Environmental & Radiological Health Sciences, Colorado State University, 1618 Campus Delivery, Fort Collins, CO 80523, USA; b Graduate School of Engineering, The University of Tokyo, Tokyo 113-8685, Japan; c Research and Development Group, Toyo Sugar Refining Co. Ltd., Tokyo 103-0046, Japan

**Keywords:** Cytotoxicity, Flavonoid, Quercetin, Isoquercetin, Rutin

## Abstract

The flavonoids quercetin, and its natural glycosides isoquercetin and rutin, are phytochemicals commonly consumed in plant-derived foods and used as a food beverage additive. Semi-synthetic maltooligosyl isoquercetin, monoglucosyl rutin and maltooligosyl rutin were developed by synthetic glycosylation to improve their water solubility for food and other applications. Using a system of Chinese hamster ovary (CHO) cells, this study examined the differences in cytotoxic responses induced by short and continuous exposure of natural and synthetic flavonoids. By assessing cell viability after short term exposure and clonogenicity with continuous exposure under various flavonoids, quercetin aglycone is confirmed to be the most cytotoxic flavonoids, and heavily glucosylated maltooligosyl rutin was the least cytotoxic. The other heavily glucosylated maltooligosyl isoquercetin showed intermediate cytotoxicity and similar toxicity as isoquercetin.

**Specifications Table**

**Table:** 

Subject area	*Biology*
More specific subject area	*Toxicology*
Type of data	*Graph*
How data was acquired	*Microscopic analysis*
Data format	*Analyzed*
Experimental factors	*No pre-treatment*
Experimental features	*Viability test by trypan blue dye exclusion and clonogenic survival test by colony formation assays*
Data source location	*Not applicable*
Data accessibility	*The data are supplied with this article*

**Value of the data**

• Immediate exposure to the natural flavonoids, quercetin, isoquercetin, and rutin, and the manufactured flavonoids, maltooligosyl isoquercetin, and monoglucosyl and maltooligosyl rutin, demonstrated that only the natural flavonoid quercetin induced cytotoxicity after 24 h of exposure.
• Continuous induction of cytotoxic responses by the natural flavonoids and semi-synthetic flavonoids confirmed that the natural flavonoids elicit greater cytotoxic responses than semi-synthetic flavonoids.
• Quercetin aglycone induced the highest cellular toxicity in the two assays we tested.
• Maltooligosyl rutin exhibited the least cytotoxicity.
• Maltooligosyl isoquercetin did not show reduced cytotoxicity compared to isoquercetin.

## 1 Data

**Fig. 1:**
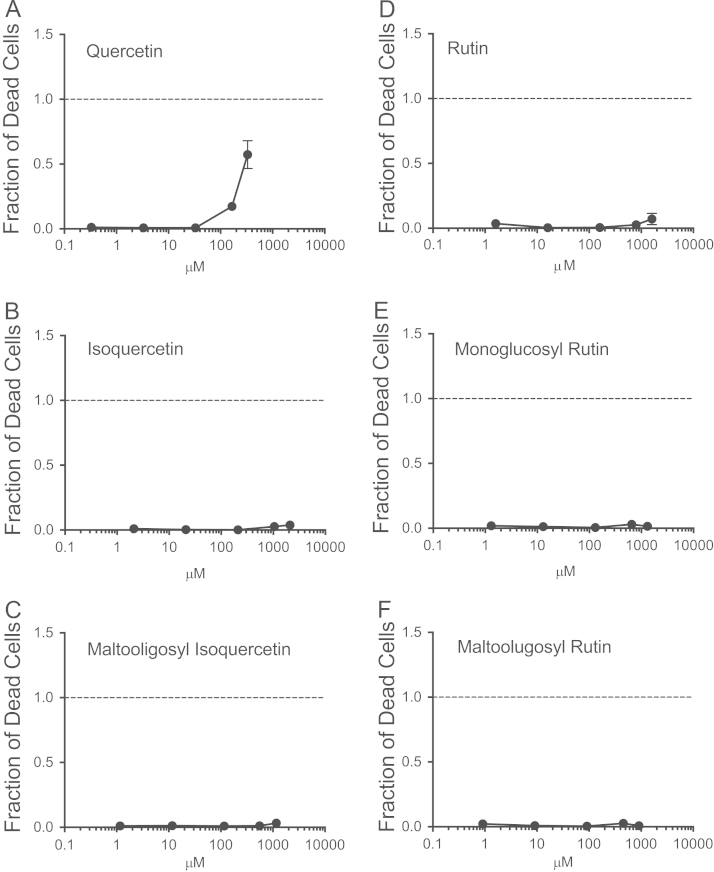
Cell viability by trypan blue dye exclusion assay. Cells were exposed to flavonoids for 24 h. (A) Quercetin, (B) iosoquercetin, (C) maltooligosyl isoquercetin, (D) rutin, (E) monoglucosyl rutin, and (F) maltooligosyl rutin. Error bars indicate standard error of the means.

Cell viability assessment was based on quantitative analysis of trypan blue dye exclusion after 24 h of exposure to the flavonoids. Quercetin showed approximately 15% cell death in 165 µM concentration and approximately 57% cell death in 330 µM concentration (Fig. 1A). In contrast, cells treated with isoquercetin, rutin, monoglycosyl rutin, maltooligosyl isoquercetin and maltooligosyl rutin did not show drastic cell death at the same or higher concentration (Fig. 1B–F). Comparison of the flavonoid treatments indicates that exposure to quercetin resulted in the most pronounced cytotoxicity.

**Fig. 2:**
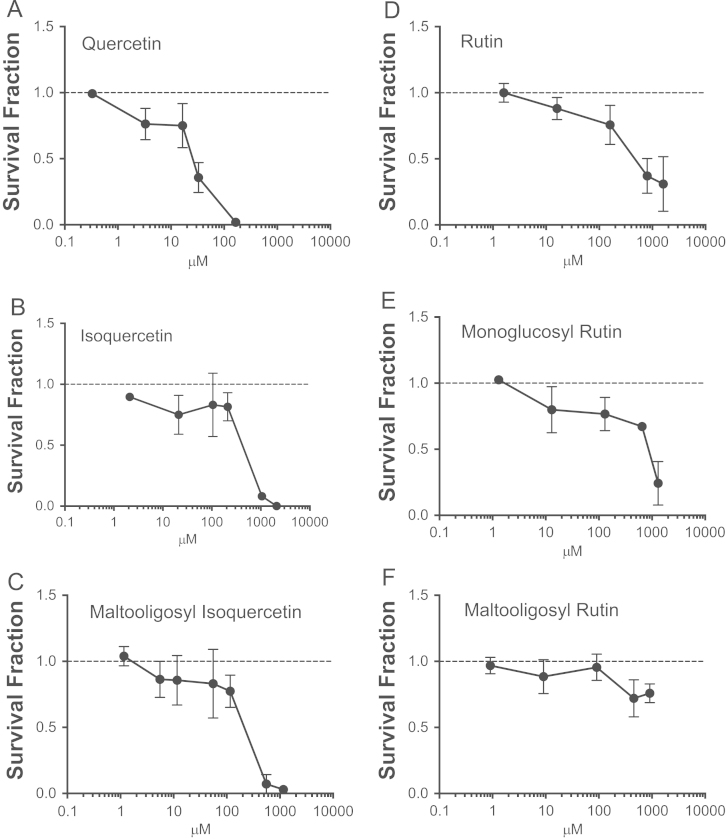
Clonogenic test by colony formation assay. Cells were exposed to flavonoids during colony formation for 7 days. (A) quercetin, (B) isoquercetin, (C) maltooligosyl isoquercetin, (D) rutin, (E) monoglucosyl rutin, (F) maltooligosyl rutin. Error bars indicate standard error of the means.

During colony formation, treatments with flavonoids were added continuously. Higher concentrations of all flavonoids showed a loss of clonogenicity. Under quercetin treatment, 50% of the cells were killed at concentrations of 20 and 30 µM (Fig. 2A). More than 50% of the cells were killed under treatments with isoquercetin, maltooligosyl isoquercetin, and rutin at an approximate concentration of 500 µM (Fig. 2B–D). Half of the cells were killed under monoglucosyl rutin treatment at approximately 700 µM (Fig. 2E). However, cells exposed to maltooligosyl rutin did not reach 50% cell death, even at the concentration of 910 µM (Fig. 2F). This further confirmed that quercetin is the most cytotoxic natural flavonoid and indicated that maltooligosyl rutin is the least cytotoxic.

## 2 Experimental design, materials and methods

Detailed experimental materials including the chemical structures of flavonoids were described in the previous publications [1], [2], [3].

### 2.1 Cell culture

CHO10B2 (CHO wild type) cells were maintained in culture in minimum essential medium (MEM-α, Gibco, Grand Island, NY), supplemented with heat inactivated 10% fetal bovine serum (FBS, Sigma, St. Louis, MO), and 1% antibiotics and antimycotics (Gibco) in a humidified incubator at 37 °C and 5% CO_2_.

### 2.2 Flavonoids

Quercetin, isoquercetin, maltooligosyl isoquercetin, rutin, monoglucosyl rutin and maltooligosyl rutin were developed and provided by the Toyo Sugar Refining Co., Ltd. (Tokyo, Japan).

### 2.3 Colony formation assay

Trypsinized CHO cells were plated on P-60 dishes to obtain approximately 100 colonies per dish. Cells were treated with various dosages of natural and semi-synthetic flavonoids during colony formation. After a 7-day incubation period, cells were washed in 0.9% (w/v) sodium chloride and fixed in 100% ethanol, then stained with 0.1% (w/v) crystal violet dye (Sigma). Macroscopic colonies containing more than 50 cells (i.e., those formed from seeded cells that did not undergo a mitotic cell death following flavonoid exposure) were marked as survivors [4]. All studies were conduced as independent experiments for a minimum of three replicates for each endpoint.

### 2.4 Trypan blue dye exclusion assay

Cells were exposed to various concentrations of flavonoids for 24 h. After washing with PBS, trypan blue was added to the cells (final concentration was 0.004%). The dye exclusion test was carried out under Zeiss Axiovert S100 inverted microscope (Carl Zeiss, Jena, Germany) [5]. Blue stained cells were counted as dead cells. At least 200 cells were scored per data point. All studies were conducted as independent experiments for a minimum of three replicates for each endpoint.
